# Prevalence of Wheat/Gluten-Related Disorders and Gluten-Free Diet in Paraguay: An Online Survey-Based Study

**DOI:** 10.3390/nu13020396

**Published:** 2021-01-27

**Authors:** Noé Ontiveros, Raúl Emilio Real-Delor, José Antonio Mora-Melgem, Carlos Eduardo Beltrán-Cárdenas, Oscar Gerardo Figueroa-Salcido, Marcela de Jesús Vergara-Jiménez, Feliznando Isidro Cárdenas-Torres, Lilian Karem Flores-Mendoza, Jesús Gilberto Arámburo-Gálvez, Francisco Cabrera-Chávez

**Affiliations:** 1 Clinical and Research Laboratory (LACIUS, URS), Department of Chemical, Biological, and Agricultural Sciences (DC-QB), Division of Sciences and Engineering, University of Sonora, Navojoa 85880, Sonora, Mexico; noe.ontiveros@unison.mx (N.O.); lilian.flores@unison.mx (L.K.F.-M.); 2 Faculty of Medicine, Itapúa National University, Itapúa 070114, Paraguay; raulemilioreal@gmail.com; 3 Nutrition Sciences Postgraduate Program, Faculty of Nutrition Sciences, University of Sinaloa, Culiacán 80019, Sinaloa, Mexico; joseantoniomoramelgem@gmail.com (J.A.M.-M.); carlos.1.beltran.uacng@uas.edu.mx (C.E.B.-C.); mjvergara@uas.edu.mx (M.d.J.V.-J.); feliznando@uas.edu.mx (F.I.C.-T.); 4 Postgraduate in Health Sciences, Division of Biological and Health Sciences, University of Sonora, Hermosillo 83000, Sonora, Mexico; gerardofs95@hotmail.com

**Keywords:** gluten-related disorders, celiac disease, wheat allergy, non-celiac gluten sensitivity, gluten-free diet

## Abstract

Gluten-related disorders (GRDs) are increasing around the world, but their magnitude and relevance remain unknown in most Latin American countries. Thus, an online survey was conducted to estimate the prevalence of GRDs as well as adherence to a gluten-free diet (GFD) in Paraguayan adult population. There were 1058 individuals surveyed using a validated questionnaire (response rate of 93.9%). The self-reported prevalence rates were as follows (95% CI): gluten sensitivity (GS), 10.30% (8.53–12.29); non-celiac GS (NCGS), 5.19% (3.94–6.71); physician-diagnosed celiac disease (PD-CD), 3.11% (2.15–4.35); wheat allergy (WA), 2.07% (1.30–3.13); and adherence to GFD, 15.69% (13.55–18.02). Excluding CD, more women than men met the criteria for GRDs, adverse food reactions, and GFD (*p* < 0.05). Eight respondents reported the coexistence of NCGS with PD-CD and/or WA. Most cases on a GFD indicated medical/dietitian advice for following the diet (68.07%). Non-self-reported GS individuals indicated weight control (46.4%) and the notion that the GFD is healthier (20.2%) as the main motivations for following the diet. GRDs are not uncommon in Paraguayan adult population. It seems that there is awareness about GRDs and the GFD, but training about the diagnosis of GRDs is desirable because of the informed overlapping diagnoses of CD or WA with NCGS. Future studies involving face-to-face interviews are necessary.

## 1. Introduction

The most frequently reported wheat/gluten-related disorders (GRDs) are celiac disease (CD), wheat allergy (WA), and non-celiac gluten sensitivity (NCGS) [1]. CD is a T-cell mediated enteropathy with auto-immune characteristics [2], while anti-wheat IgE antibodies are the hallmark of WA [3]. Regarding NCGS, the most accepted mechanisms underlying it have been associated with the innate immune system, and it is not recognized as a strict enteropathy [4]. The wheat/gluten-free diet (GFD) is the only accepted treatment for these three conditions. Although it is intended as a treatment, some people follow the GFD without a previous diagnosis of a GRD and/or medical/dietitian advice [5,6]. The gold standards for diagnosing NCGS and WA involve the evaluation of specific biomarkers and clinically controlled wheat/gluten challenges [2,7]. The gold standard for CD diagnosis is the gastrointestinal endoscopy with intestinal biopsies while the patient is on a diet with gluten [2]. These clinical practices make it difficult to estimate the gold standard-based prevalence of GRDs at a population level. Alternatively, survey-based studies have been successfully applied in several populations in order to estimate not only the prevalence of GRDs, but also the prevalence of adherence to a GFD [5,6,8,9,10,11,12,13,14]. Despite this, the magnitude and relevance of GRDs and adherence to a GFD in Latin America remains to be established. In an attempt to contribute to the knowledge on the prevalence of these disorders and their treatment in Latin America, our research group carried out survey studies in this region [5,6,11,12,13,14]. These survey studies were carried out through face-to-face interviews, but the current COVID-19 (SARS-COV-2) pandemic has hampered the collection of data using such an approach. Online platforms have been widely utilized for collecting data without the need for face-to-face interaction, and seem to be the approach of choice for overcoming the SARS-COV-2/COVID-19 health problems for continuing to carry out survey studies. Paraguay is a South American country where some studies focused on CD have been carried out [15,16,17] and where the consumption of wheat has been increasing [18], but there is a lack of epidemiological data about GRDs and adherence to a GFD. Thus, the aim of the present study was to estimate the self-reported prevalence rates of GRDs as well as adherence to a GFD in a Paraguayan adult population utilizing an online platform.

## 2. Materials and Methods

### 2.1. Questionnaire and Population Survey

A validated self-administered questionnaire was utilized [13]. The questionnaire included questions regarding demographic data, symptoms triggered after the consumption of wheat/gluten, the type and onset of the symptoms, physician diagnosis of GRDs and other diseases, and adherence to a GFD and gluten avoidance.

A digital version of the questionnaire was developed using the SurveyMonkey platform (San Mateo, CA, USA). The hyperlinks generated were sent through text messages and emails to the Paraguayan population from the Itapúa Department. The following inclusion criteria were used: (1) Paraguayan individuals who consented to participate in the present study, (2) who were ≥18 years old, and (3) who could read and answer the questionnaire by themselves. For informed consent, the first page of the survey was designed to display a summary of the study, and participants had to agree to participate in the study in order to be able to answer the questionnaire. Questionnaires with incomplete demographic data were excluded. All of the data were collected from 17 October to 20 November 2020.

### 2.2. Classification of the Participants

**Figure 1:**
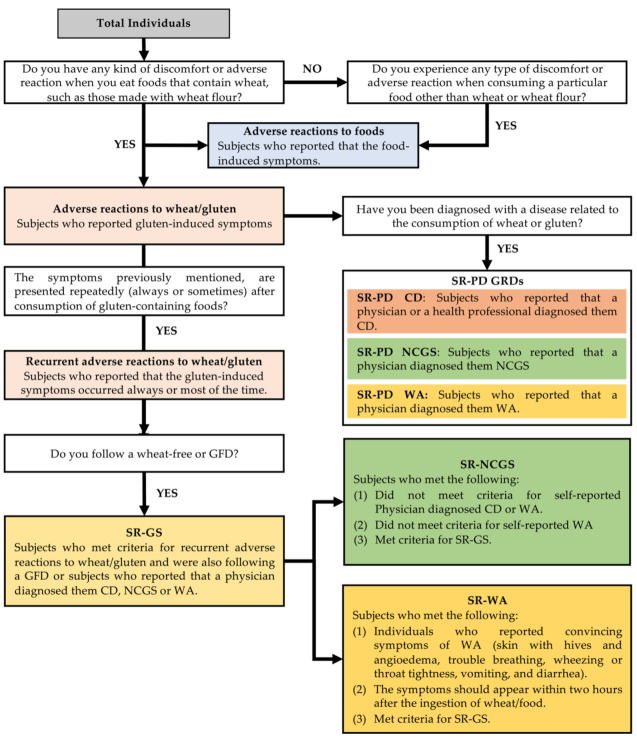
Algorithm and criteria to classify respondents. SR-GS—self-reported gluten-sensitivity; GFD—gluten-free diet; SR-PD—self-reported physician-diagnosed; GRDs—gluten-related disorders; CD—celiac disease; NCGS—non-celiac gluten-sensitivity; WA—wheat allergy.

Participants were classified in one of eight categories, as previously described [5,6,11,12,13,14], namely adverse reactions to foods, adverse reactions to wheat/gluten, self-reported gluten-sensitivity (SR-GS), self-reported physician-diagnosed WA (SR-PD WA), self-reported physician-diagnosed CD (SR-PD CD), self-reported WA (SR-WA), self-reported physician-diagnosed NCGS (SR-PD NCGS), and self-reported NCGS (SR-NCGS). Figure 1 shows the criteria used to classify participants.

### 2.3. Statistical Analysis and Ethical Issues

The statistical software GraphPad Prism Version 5.0 (GraphPad Software, San Diego, CA, USA) was utilized. A set of descriptive statistics (total numbers, percentages, odds ratio, and 95% confidence intervals) was used for analyzing the categorical variables. A two-tailed Fisher’s exact test was performed to determine associations. The mean differences between groups were determined using Student’s *t*-test. A *p* value < 0.05 was considered to be statistically significant. The prevalence rates were estimated using the OpenEpi software version 3.03a (Atlanta, GA, USA) and reported per 100 inhabitants (95% confidence intervals). The sample size was calculated considering an infinite population, a level of confidence interval of 95%, an expected prevalence of GRDs of 7%, and a precision of 2%. With these parameters, a sample size of at least 626 individuals was considered representative. The Ethics Review Board of the Faculty of Medicine of the National University of Itapúa reviewed and approved the study protocol. The ethical guidelines established by the Declaration of Helsinki were considered.

## 3. Results

### 3.1. Demographic and Clinical Characteristics

A total of 1127 individuals followed the link to participate in the study. Of these individuals, 1118 agreed to participate and answered the questionnaire (a response rate of 99.2%). Sixty individuals proportioned incomplete data and were excluded from the study (valid response rate of 93.9%; n = 1058). The respondents were mainly women (65.1%). The most common self-reported physician-diagnosed conditions were irritable bowel syndrome (8.79%), lactose intolerance (8.7%), and non-food allergy (6.9%; Table 1).

### 3.2. Prevalence Rates

The prevalence rates estimated are shown in Table 1. The general prevalence of adverse reactions to wheat/gluten was 21.26% (n = 225). Although 177 individuals reported recurrent adverse reactions to wheat/gluten or a physician diagnosis of GRDs, only 109 individuals met the criteria for SR-GS (general prevalence (95% CI), 10.30% (8.53–12.29)). Thirty-three responders reported that they had a physician diagnosis of CD (general prevalence (95% IC), 3.11% (2.15–4.35)). The general prevalence rates of WA and NCGS were 2.07% (95% CI, 1.30–3.13) and 5.19% (95% CI, 3.94–6.71), respectively. Excluding CD, all self-reported conditions were more prevalent in women than in men (*p* < 0.05; Table 2).

### 3.3. Overlapping of Physician-Diagnosed Gluten-Related Disorder

Eight respondents reported the coexistence of NCGS with either CD or WA, or with both conditions. Two of these cases had to be excluded from the prevalence estimates of GRDs because of the uncertainty of their diagnoses, and they were on a gluten-containing diet. The other six cases met the criteria for SR-GS and were re-classified. The characteristics of these cases are show in Table 3.

### 3.4. Symptoms Reported by SR-GS Cases

**Figure 2:**
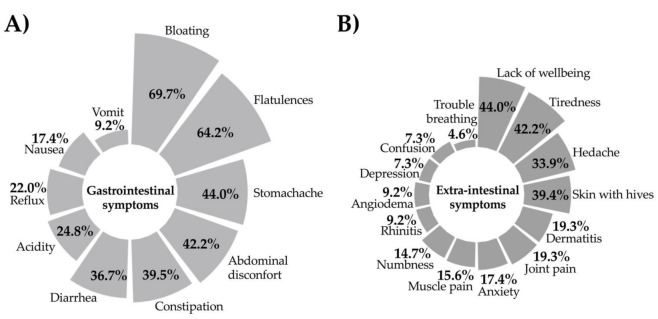
Self-reported (**A**) gastrointestinal and (**B**) extra-intestinal symptoms in SR-GS individuals (n = 109).

Among the individuals that met the criteria for SR-GS (n = 109), 95.4% (n = 104) reported gastrointestinal and 84.4% (n = 92) reported extra-intestinal symptoms. Bloating (69.7%), flatulence (64.2%), and stomachache (44.0%) were the most common gastrointestinal symptoms (Figure 2A). Lack of wellbeing (44.0%), tiredness (42.2%), and headache (33.9%) were the most common extra-intestinal symptoms (Figure 2B). Most individuals (82.6%; n = 90) reported more than one recurrent symptom, either gastrointestinal or extraintestinal, or both.

### 3.5. Gluten-Free Diet

**Figure 3:**
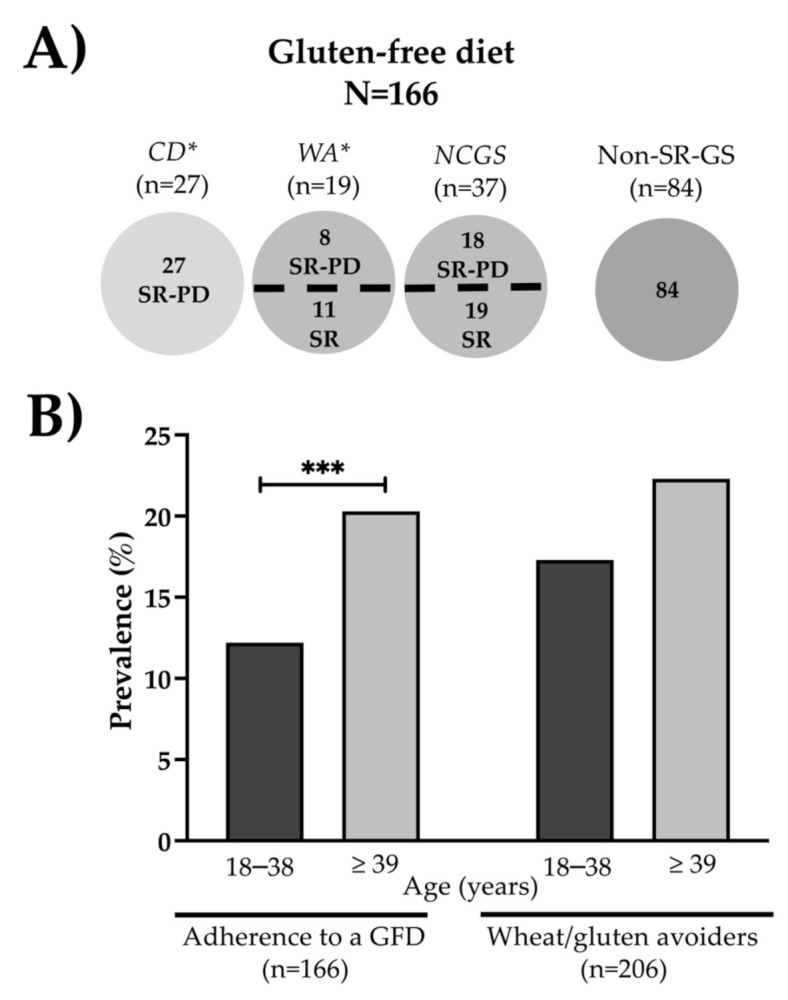
Prevalence of adherence to a GFD and wheat/gluten avoiders stratified by age. (**A**) Characteristics of individuals following a GFD. (**B**) GFD adherence and avoidance of wheat/gluten according to age. * One subject reported that a physician diagnosed her/him both CD and WA and was on a GFD. *** *p* < 0.001.

The characteristics of the individuals following a GFD are shown in Figure 3A. The prevalence of adherence to a GFD was 15.69% (95% IC, 13.55–18.02). Most CD cases (27 out of 33) were following a GFD. Half of the participants who reported that they were following a GFD were non-SR-GS cases (50.6%; n = 84; Figure 3A). Among these cases, four individuals (4.8%) reported non-recurrent adverse reactions to wheat/gluten and the other eighty individuals (95.2%) did not report any adverse reaction to gluten. Regarding those individuals who reported adverse reactions to wheat/gluten consumption (n = 225), 38.2% of them (n = 86) were following a GFD and 32.4% (n = 73) reported that they were doing their best avoiding wheat/gluten in their diets. The prevalence of wheat/gluten avoiders was 19.47% (95% IC, 17.13–21.99; n = 206), and most of these cases did not report adverse reactions to wheat/gluten (64.56%; n = 133). The prevalence of adherence to a GFD was higher in the group aged ≥39 than in the one aged 18–38 years old (20.3% vs. 12.2%; *p* < 0.001). Non-statistical differences were observed in the prevalence rates of gluten avoiders stratified by age (Figure 3B).

### 3.6. Reasons for Gluten-Free Dietary Non-Compliance and Motivations for Following a GFD

Among the SR-PD GRD cases (n = 79), 34% (n = 27) were not following a GFD. The main reasons reported for gluten-free dietary non-compliance were the mildness of the symptoms triggered after gluten ingestion (47.0%), and the low availability and high cost of gluten free products (41.2%; data not shown).

**Figure 4:**
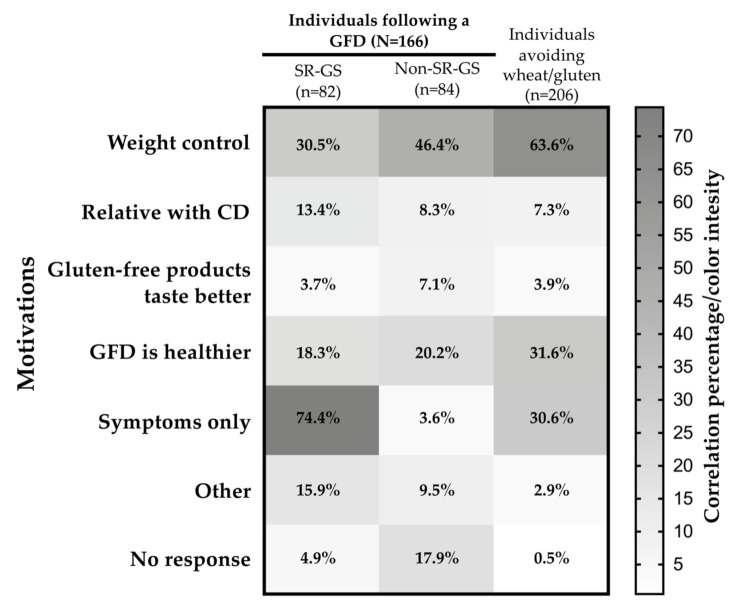
Motivations for following a GFD among SR-GS and non-SR-GS cases, or for avoiding wheat/gluten from the diet among wheat/gluten avoiders. GFD—gluten-free diet; CD—celiac disease.

Regarding who instructed the GFD, 26.8% (22 out of 82) of the SR-GS cases informed that they were following the diet without medical/dietitian advice. Some of them (74.4% (61 out of 82)) also indicated that the main motivation for following a GFD was the symptoms triggered by wheat/gluten ingestion (Figure 4). A lack of dietary counseling was also reported by 36.9% (31 out of 84) of the non-SR-GS cases. In this group, the main motivation for following the GFD was weight control (46.4% (39 out of 84); Figure 4). Similarly, among wheat/gluten avoiders (n = 206), 63.3% reported weight control as the main motivation for avoiding wheat/gluten in the diet (Figure 4).

## 4. Discussion

Carrying out survey studies under the approach of face-to-face interviews has not been viable in the year 2020 because of ethical issues related to the current COVID-19 pandemic. Fortunately, digital platforms offer the opportunity to continue generating survey-based epidemiological data. In the present study, we took advantage of the SurveyMonkey digital platform to assess the self-reported prevalence of GRDs and adherence to a GFD in a Paraguayan adult population. The results show that recurrent adverse reactions to oral wheat/gluten are common in the Paraguayan adult population (16.7%), and that the prevalence of SR-GS (10.3%) is higher than the prevalence rates reported in other Latin American countries (1.06–6.31%) [5,6,11,12,13,14] and elsewhere (0.25–3.7%) [10,19,20], considering the same definitions of SR-GS. Similarly, the prevalence rates of CD (3.11%), WA (2.07%), and NCGS (5.19%) were higher than the rates reported in other survey studies (0–1.2%, 0.24–0.79%, and 0.25–3.7%, for CD, WA, and NCGS, respectively) [5,6,11,12,13,14]. It should be noted that the prevalence rates of GRDs reported in the present study were obtained in the Paraguayan adult population that own a smartphone and have internet access, and that some GS individuals could be included in the 26.4% of the Paraguayan population that do not have internet access [21]. All previous survey studies that estimated the prevalence rates of GRDs were carried out with face-to-face interviews and the data were collected outside shopping malls, train and bus stations, and urban parks, which allow for including a very diverse population. Despite the potential limitations in the target population, our data corroborate previous findings by showing that GRDs are significantly more common in women than in men [5,6,12], and highlight that studies are required for an in-deep understanding of the molecular and/or genetic bases of the gender differences in the prevalence rates of GRDs.

It should be noted that some self-reported physician-diagnosed GRDs identified in the present study were misdiagnosed cases, as CD or WA cannot coexist with NCGS. In fact, CD and WA have to be ruled out in order to establish a proper diagnosis of NCGS [7]. Similar findings were reported in a survey study carried out in another region of Latin America [11]. In the present study, most of the overlapped diagnoses of CD with NCGS or WA were physician-diagnosed cases (6 out of 8), but only one of the cases reported that a gastroenterologist diagnosed her/his condition (NCGS). Assessing CD by non-specialist physicians is sometimes based on celiac serology only [22], but the prevalence of positive serology is almost three-fold higher than the prevalence of CD based on a formal diagnosis [23]. At the moment, the instrument utilized did not inquire about the diagnostic procedures of the GRDs reported by the participants. Although this last fact could adversely impact on drawing definitive survey-based conclusions related to the physicians’ diagnostic skills and training for carrying out the diagnosis of GRDs, the data collected are fair enough for suggesting actions for properly diagnosing GRDs. Future studies should include questions about this topic for deepening our understanding about the reasons motivating physicians for diagnosing the coexistence of NCGS with other GRDs. Overall, the results suggest that training about the diagnosis of GRDs is desirable among healthcare professionals, although there is awareness about the conditions.

The prevalence of adherence to a GFD was similar to that reported in the Chilean population (15.69 vs. 16.45%), but higher than the GFD prevalence rates estimated in other Latin American countries (5.9–7.8%) [5,6,11,12,13,14] and elsewhere (3.7%) [10,19,20]. It is common that people following a GFD are doing it without medical/dietitian advice and for reasons other than for treating a GRD, such as weight control or because they believe that the GFD is healthier than a regular one [5,6,11,14]. The results of the present study support these previous findings about the reasons for following a GFD, and also support that the mildness of the symptoms triggered is the main motivation for gluten-free dietary non-compliance in subjects that reported recurrent adverse reactions to oral wheat/gluten. However, most Paraguayan individuals (68.07%) on a GFD reported that were following the diet as a result of medical/dietitian advice. It is possible that the diagnosis of CD helps to improve compliance with a GFD and, consequently, to avoid the long-term complications associated with the condition, as almost 80% (27 out of 33) of the individuals with a physician diagnosis of CD indicated that were following the diet. This percentage is considerably higher than the 33% of adherence to a GFD among Paraguayan celiac patients reported a few years ago [16]. This potential increase in adherence to a GFD could be influenced by the activism of Paraguayan celiac associations like FUPACEL (Fundación Paraguaya de Celiacos), which promotes free monthly informative sessions for its members. It is noteworthy that the data generated about the GFD in the present digital survey study are in line with other studies carried out in Latin America that are done under the approach of face-to-face interviews [5,6,11,12,13,14]. Although some have reported a high consistency between data collected by online platforms and by face-to-face interviews [24], and for additionally contributing to the knowledge of the GRDs and adherence to a GFD in a Paraguayan population, future studies involving face-to-face interviews and targeting a more diverse population than the one addressed in the present study are necessary.

## 5. Conclusions

Because of the current COVID-19 pandemic, it is unfeasible to carry out survey studies under the approach of face-to-face interviews, and the most widely-applied digital survey tool was instead utilized to estimate the prevalence rates of GRDs and adherence to a GFD in an adult Paraguayan population in this study. The results show that GRDs are not uncommon among the adult Paraguayan population, and that there is a high proportion of subjects following a GFD with medical/dietitian advice, although some of them are following the diet for reasons that are not for treating a GRD. Furthermore, the results suggest that there is awareness about GRDs among health professionals, but that training about diagnoses is desirable. Future survey studies in the Paraguayan population under the approach of face-to-face interviews are guaranteed.

## Figures and Tables

**Table 1:** Demographic and clinical characteristics of the studied population.

Variable *	%	n (Male/Female)
Gender	34.9/65.1	369/689
Non-food allergy	6.90	73
IBS **	8.79	93
Colitis	1.42	15
Lactose intolerance	8.70	92
Psychiatric disease	1.51	16
Food intolerance	1.70	18
Food allergy	3.21	34
Eating disorsers	1.42	15
Diabetes mellitus	4.73	50
Gastrointestinal cancer	0.09	1

* Self-reported physician-diagnosed diseases were considered for the analysis. ** Irritable bowel syndrome.

**Table 2:** Self-reported prevalence rates of adverse reactions to food and GRDs.

Assessment	(+) Cases *	Mean Age in Years (Range)	Prevalence by Gender % (95% CI)	*p* Value	General Prevalence % (95% CI)
Adverse reactions to food	Total = 337	40.9 (19–84)	M 19.24% (15.34–23.64) F 38.60% (34.95–42.36)	<0.0001	31.85% (29.05–34.76)
	M = 71				
	F = 266				
Adverse reactions to wheat/gluten	Total = 225	42.5 (19–84)	M 9.21% (6.46–12.64) F 27.72% (24.41–31.23)	<0.0001	21.26% (18.84–23.86)
	M = 34				
	F = 191				
Recurrent adverse reactions to wheat/gluten	Total = 177	42.6 (19–84)	M 6.23% (3.99–9.20) F 22.35% (19.29–25.65)	<0.0001	16.72% (14.53–19.12)
	M = 23				
	F = 154				
(a) SR-GS *	Total = 109 †	42.89 (21–84)	M 4.06% (2.29–6.61) F 13.64% (11.17–16.43)	<0.0001	10.30% (8.53–12.29)
	M = 15				
	F = 94				
(b) Celiac disease	Total = 33	42.84 (22–84)	M 1.89% (0.76–3.87) F 3.77% (2.48–5.48)	0.1361	3.11% (2.15–4.35)
	M = 7				
	F = 26				
(c) Wheat allergy	Total = 22	44.0 (21–84)	M 0.27% (0.006–1.50) F 3.04% (1.89–4.62)	0.0012	2.07% (1.30–3.13)
	M = 1				
	F = 21				
(d) SR-NCGS **	Total = 55	43.2 (21–66)	M 1.89% (0.76–3.87) F 6.96% (5.18–9.13)	0.0002	5.19% (3.94–6.71)
	M = 7				
	F = 48				
Adherence to GFD ***	Total = 166	42.7 (18–84)	M 8.13% (5.55–11.4) F 19.73% (16.83–22.91)	<0.0001	15.69% (13.55–18.02)
	M = 30				
	F = 136				
Avoidance of wheat/gluten-containing foods	Total = 206	40.0 (18–77)	M 15.17% (11.67–19.25) F 21.77% (18.74–25.04)	0.0114	19.47% (17.13–21.99)
	M = 56				
	F = 150				

* SR-GS—self-reported gluten sensitivity; ** SR-NCGS—self-reported non-celiac gluten sensitivity; *** GFD—gluten-free diet. † one subject reported that a physician diagnosed her/him CD and WA.

**Table 3:** Characteristics of the physician-diagnosed individuals who reported the coexistence of NCGS with other gluten-related disorders.

Individual	Diagnosed by	Diagnosed Disorder	Following a GFD	Recurrent Symptoms to Wheat/Gluten	Excluded or Re-Classified	Criteria for Exclusion/Inclusion
I-1	Physician and dietitian	NCGS CD	Yes	Yes	Re-classified	CD or WA does not co-exist with NCGS. The participant met the criteria for SR-GS and CD.
I-2	Physician and dietitian	NCGS CD	Yes	Yes	Re-classified	CD or WA does not co-exist with NCGS. The participant met the criteria for SR-GS and CD.
I-3	Profesor	NCGS WA	No	Yes	Excluded	CD or WA does not co-exist with NCGS. The participant neither met criteria for WA nor SR-GS and WA.
I-4	Profesor	NCGS WA	No	No	Excluded	CD or WA does not co-exist with NCGS. The participant neither met criteria for WA nor SR-GS and WA.
I-5	Gastroenterologist and dietitian	NCGS WA	Yes	Yes	Re-classified	CD or WA does not co-exist with NCGS. The participant met the criteria for SR-GS and WA
I-6	Endocrinologist	NCGS WA	Yes	Yes	Re-classified	CD or WA does not co-exist with NCGS. Met the criteria for SR-GS and WA
I-7	Physician	NCGS CD	Yes	Yes	Re-classified	CD or WA does not co-exist with NCGS. The participant met the criteria for SR-GS and CD
I-8	Dermatologist	NCGS CD WA	Yes	Yes	Re-classified	CD or WA does not co-exist with NCGS. The participant met the criteria for SR-GS, CD and WA

## Data Availability

The data presented in this study are available within the article.
